# Prefrontal NAA and Glx Levels in Different Stages of Psychotic Disorders: a 3T ^1^H-MRS Study

**DOI:** 10.1038/srep21873

**Published:** 2016-02-23

**Authors:** Edith Liemburg, Anita Sibeijn-Kuiper, Leonie Bais, Gerdina Pijnenborg, Henderikus Knegtering, Jorien van der Velde, Esther Opmeer, Annerieke de Vos, Jozarni Dlabac-De Lange, Lex Wunderink, André Aleman

**Affiliations:** 1 Department of Neuroscience, and BCN Neuroimaging Center, University of Groningen, University Medical Center Groningen, Groningen, the Netherlands; 2 Rob Giel Research Center, University of Groningen, University Medical Center Groningen, Groningen, the Netherlands; 3 Lentis Research, Center for Mental Health, Groningen, the Netherlands; 4 Department of Psychology, University of Groningen, Groningen, the Netherlands; 5 Department of Clinical Psychology and Experimental Psychopathology, Faculty of Behavioral and Social Sciences, University of Groningen, Groningen, the Netherlands; 6 Department of Education and Research, Friesland Mental Health Care Services, Leeuwarden, the Netherlands

## Abstract

H-Magnetic Resonance Spectroscopy (^**1**^H-MRS) can offer insights in various neuropathologies by measuring metabolite levels in the brain. In the current study we investigated the levels of glutamate + glutamine (Glx, neurotransmitter and precursor) and N-Acetyl Aspartate + glutamic acid (NAA + NAAG; neuronal viability) in the prefrontal cortex of patients with a psychotic disorder and people at Ultra High Risk (UHR) for psychosis. A ^**1**^H-MRS spectrum was acquired in 31 patients with a recent onset psychotic disorder and 60 with a chronic state, 16 UHR patients and 36 healthy controls. Absolute metabolite levels were calculated using LCModel with a reference water peak. Groups were compared while taking into account age and partial volume effects. Moreover, we investigated associations with positive and negative symptoms, duration of illness, and antipsychotic treatment in patients. The most notable finding is that chronicity of schizophrenia was related to decreased levels of Glx and NAA. On the other hand, although on an exploratory note, UHR showed increased levels of prefrontal Glx and NAA levels with increasing age. Our results may indicate an initial Glx and NAA increase and subsequent decrease during illness progression that may be related to the neurotoxic effects of glutamate.

The severity and pattern of symptoms associated with psychotic disorders typically evolve over time. Before the onset of a clinical psychosis, persons often have attenuated psychotic symptoms, and show a marked decline in social and occupational function^1^. This state has been referred to as being at Ultra High Risk (UHR) for psychosis^2^. Starting from this prodromal phase, progressive changes in neuropathology may occur^3^. Studies have shown progressive loss of brain tissue, including the prefrontal cortex (PFC)^4^,^5^. In addition, decreased lateral prefrontal activation (‘hypofrontality’) and increased medial frontal activation have often been regarded as a core feature of schizophrenia^6^,^7^,^8^ and have also been observed in first episode psychosis^9^.

Neurotransmitters play an important role in the neuropathology of psychotic disorders. Historically, overstimulation of dopaminergic D2 receptors in certain parts of the brain has been considered to lead to hallucinations, delusions, and other positive symptoms^3^,^10^. A concurrent prefrontal hypodopaminergia, mediated by abnormalities in serotonergic and GABAergic function, may contribute to negative and cognitive symptoms^10^. Glutamate also seems to play an important role in psychotic disorders; decreased levels of prefrontal glutamate may be involved in the pathogenesis of negative symptoms and cognitive impairments^3^,^11^. Disturbances in glutamate may cause an ongoing pathophysiological process that causes brain tissue loss during illness progression^12^,^13^. The NMDA-hypofunction hypothesis states that glutamate receptor dysfunction may cause toxically elevated levels of glutamate in early phases of psychotic disorders that result in tissue loss, which may eventually cause decreased glutamate levels^14^.

Proton Magnetic Resonance Spectroscopy (^**1**^H-MRS) is a non-invasive MR technique that can offer insights in altered metabolite levels caused by various neuropathologies^14^,^15^,^16^,^17^. Using conventional MRS at magnetic field strengths of 1.5 to 4 T, it is challenging to separate the glutamate and glutamine signals and thus the combined signal Glx is often measured. N-Acetyl Aspartate (NAA) is the second most abundant metabolite in the human brain and emits the strongest signal in ^**1**^H-MRS. Its abundance may reflect neuronal health and neuronal metabolism^15^,^16^,^18^. In schizophrenia, lower levels of glutamate and NAA may provide insight in loss of gray and especially white matter tissue that may occur due to neurotoxicity^14^,^18^,^19^.

Despite studies that fail to show abnormalities in Glx and NAA in both patients and UHR subjects^12^,^16^,^19^,^20^, many studies have shown that NAA levels are lower in the (lateral) prefrontal cortex of patients with schizophrenia^14^,^16^,^17^,^18^, and also in UHR subjects^21^. Moreover, an early age of onset of psychosis has also been related to lower NAA levels^19^. Studies on Glx have reported mixed findings^19^, but the general picture is that Glx levels are increased in the lateral PFC of recently ill patients and in the medial PFC during all stages of illness, and that levels may decrease with increasing age or longer durations of illness^13^,^16^,^22^,^23^. While prefrontal Glx and NAA levels decrease with age in healthy subjects, this decrease has been suggested to be more pronounced in patients with schizophrenia^12^,^16^,^17^.

To date, the glutamate and NAA levels have mostly been investigated in gray matter, while white matter may be more strongly related to neurodevelopmental changes in schizophrenia^4^. Moreover, there are limited studies that investigated UHR subjects or the effect of duration of psychosis^12^,^23^. Moreover, most studies have not investigated the relationship of altered metabolite levels with symptoms and antipsychotic medication^12^; such relationships are plausible, however^3^,^11^,^24^. The few available studies on the effect of antipsychotics have shown both increases and decreases of metabolite levels, that may be dependent on type, dose or duration of treatment^13^,^16^,^22^, but reduced Glx and NAA levels have been related to stronger D2 antagonists^16^,^17^,^19^. Several studies have shown a negative relation between Glx and NAA levels and negative symptoms^13^,^16^,^19^. Finally, many studies have investigated relative metabolite ratios, while the reference metabolite creatine may also be altered in schizophrenia^18^ or have omitted correction for differences in tissue content in the voxel (partial volume correction)^12^,^16^.

In the current study, we aim to investigate lateral prefrontal white matter Glx and NAA levels in recent onset patients and chronic patients as well as subjects with an UHR state compared to matched healthy controls. We tested the hypothesis whether subjects with UHR would have increased levels of Glx and decreased levels of NAA and whether psychotic patients have lower Glx and NAA levels than healthy controls, related to duration of illness. Moreover, we investigated whether Glx and NAA levels may be related to antipsychotic use, and positive and negative symptoms. Finally, we investigated absolute levels (instead of ratios) and we corrected for cerebral spinal fluid (CSF) and gray matter (GM) volume.

## Materials and Methods

### Subjects

We included 31 patients with a recent onset (duration of illness <2 years) and 60 with a chronic psychotic disorder (schizophrenia, schizoaffective disorder, psychosis NOS, delusional disorder), 16 UHR patients and 36 matched controls (16 matched to the UHR and 20 to the patients from one other study). All patients were recruited from mental health institutions in the northern parts of the Netherlands for four different studies; we pooled all pretreatment baseline data to achieve maximum statistical power. No ^**1**^H-MRS findings from these studies have been published before. The first study investigated the effects of treatment with aripiprazole compared to risperidone on negative symptoms (EUDRA-CT: 2007-002748-79)^25^. The second functional magnetic resonance imaging (fMRI) study was part of a double-blind multicenter randomized controlled trial investigating the effect of repetitive transcranial magnetic stimulation (rTMS) on negative symptoms (Dutch Trial Registry: NTR1261)^26^. The third was a multicenter randomized controlled trial on a brief psychosocial intervention to improve insight of schizophrenia in patients with poor insight (NL2714604209)^27^. A fourth study investigated neural correlates of cognitive-emotional processing in an UHR sub-sample, i.e. help-seeking young people with an Ultra High Risk to develop psychosis, which was part of a larger study^28^.

Inclusion criteria for these studies included having a diagnosis within the DSM-IV classification schizophrenia and other psychotic disorders (measured with the Mini-International Neuropsychiatric Interview (MINI)^29^ or Schedules for Clinical Assessment in Neuroscience interview (SCAN)^30^; patients in the first three studies), an age older than 18 years and being able to give informed consent. UHR subjects should score positive on the Comprehensive Assessment of At Risk Mental State interview (CAARMS)^2^. Exclusion criteria included having a co-morbid neurological disorder, not having sufficient mastery of the Dutch language, and specific MRI exclusion criteria, including pregnancy or possibility thereof, red ink tattoos, metal implants in the body and claustrophobia. All patients signed written informed consent before the scanning session, after the procedure had been fully explained. All study protocols were all approved by the medical ethical board of the University Medical Center Groningen (METC; UMCG), except the UHR study that was approved by the Mental Healthcare Research Ethics Committee (METIGG). All procedures were carried out according to the declaration of Helsinki.

Education was measured according to the Verhage system, with a scale ranging from 1 = primary school to 8 = university^31^. Symptom severity was measured by the Positive and Negative Syndrome Scale (PANSS)^32^. Based on antipsychotic dose the haloperidol equivalents were calculated^33^. Using no antipsychotics was defined as 0 mg haloperidol equivalents. An overview of the subject characteristics is given in Table 1. There was a significant difference in age between the groups, possibly caused by a higher age of the chronic sample, a significant difference of negative symptoms, caused by less negative symptoms in the UHR group and chronic patients also used higher doses of antipsychotic medication. Other effects were non-significant. In the recent-onset patients, 77% had a diagnosis of schizophrenia or schizoaffective disorder. The other patients also had a schizophrenia spectrum psychotic disorder; due to their short duration of illness (<6 months) a diagnosis of schizophrenia could not be confirmed. The duration of illness varied from 0–30 years. Patients and UHR had on average mild to moderately severe symptoms (2–3 on average per PANSS item).

### MR acquisition

All scans were acquired in the Neuroimaging Center of the University Medical Center Groningen (UMCG) in Groningen. Scans were acquired using a 3T Philips Intera (Best, the Netherlands) equipped with a synergy SENSE eight-channel head coil. ^1^H-MRS single-voxel spectroscopy was used to assess proton metabolites in the white matter of the left lateral prefrontal cortex with an 8 cm^3^ voxel. The voxel was placed in line with the genu of the corpus callosum on the anterior side and oriented in the same line as the corpus callosum and the falx cerebri, inclusion of white matter was maximized. See Supplementary Fig. S1 for reference. This examination was carried out using Point Resolved Spectroscopy (PRESS) sequence of 5 minutes, with one 90° and two 180° pulses, and water suppression with a selective 140 Hz RF pulse and a subsequent RF inversion pulse. This was the standard protocol when the data acquisition started. Automated first-order B0 shimming at the ROI was performed prior to MRS. Spectra were recorded within the following parameters: TE = 144 ms, TR = 2000 ms, samples = 1024, bandwidth = 2000 Hz, VOI = 20 × 20 × 20 mm, signal averages (NSA) = 128. For anatomical reference and localization of the MRS voxel, a T1-weighted image (160 slices; isotropic voxels of 1 mm; TR 25 ms; TE 4.6 ms; α 30°; FOV 256 mm) covering the whole brain was acquired.

## Data Analysis

The spectral data of the glutamate + glutamine (referred to as Glx) peak and the NAA + glutamic acid (NAA + NAAG; referred to as NAA) peak were analyzed with LCModel^34^. Absolute metabolite levels were determined by scaling based on the unsuppressed water peak. Data were excluded if metabolite concentrations had an estimated standard deviation higher than 20% of the estimated concentration (Cramer-Rao bounds) or deviated more than 3 standard deviations (SDs) from the group mean. The anatomical scan used for the voxel placement was segmented using SPM8 (FIL Wellcome Department of Imaging Neuroscience, London, UK). The segmented scans were used to determine the gray matter (GM) and cerebrospinal fluid (CSF) content of the spectroscopy voxel. Correct localization of the voxel on the segmented scans was confirmed by checking a picture of the voxel placement acquired during scanning. The extracted percentages of GM and CSF were added to subsequent regression analyses to correct for partial volume effects.

Statistical analysis was performed with SPSS 20 (IBM Inc. New York, USA). First demographical data were compared between groups (α = 0.05). Because of non-normality of the data, age, education, haloperidol equivalents and symptoms were compared using a Kruskal-Wallis or Mann-Whitney U test, and gender with a Chi-square test for independence.

Next, the average and standard deviation of the Glx and NAA level were determined, together with 95% confidence interval (Cramer-Rao bounds) of measurement precision. Both were normally distributed. Moreover, the correlation between both metabolites was determined. To select confounding variables, the correlations between metabolites and age, gender, GM and CSF content were also calculated.

First, the four groups of subjects (controls, UHR, recent onset and chronic patients) were compared with an one-way ANOVA using post-hoc tests. Next, an ANCOVA was performed with significant covariates (age, GM and CSF).

Subsequently, the effect of age, and positive and negative symptoms in patients (recent onset and chronic sample combined) and UHR subjects was investigated using linear regression for both groups separately. Duration of illness and antipsychotic load (haloperidol equivalents) were investigated in patients. In case of a significant effect, age, GM and CSF were added as covariates to all models, but age was left out in the regression of age and duration of illness, given their strong intrinsic association to avoid collinearity.

## Results

**Figure 1:**
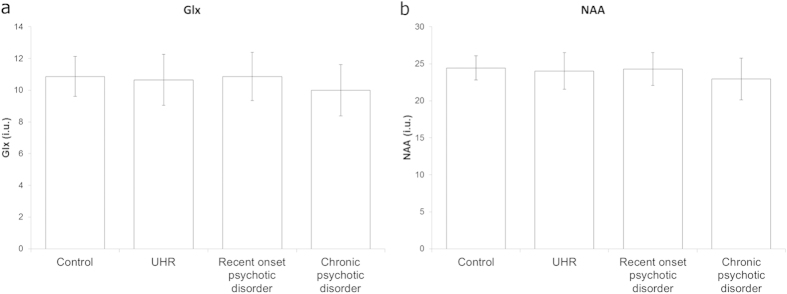
Bar graphs of average metabolite levels and standard deviation per group. Left (**a**) Glx and right (**b**) NAA for recent-onset and chronic psychosis, UHR and healthy subjects, error bars represent standard deviations. ANOVAs and post-hoc tests showed that chronically ill patients have significantly lower Glx and NAA levels.

Average metabolite levels per group are shown in Fig. 1. One chronic patient was excluded because metabolite concentrations had a standard deviation higher than 20% of the estimated concentration, and one patient and one UHR subject were excluded because the metabolite concentrations deviated more than 3 standard deviations (SDs) from the group mean. LC Model determined an average NAA level of 23.8 i.u. (institutional units; SD = 2.4), 95% CI = [23.3–24.5]. For Glx, the measured level was 10.5 i.u. (SD = 1.6), 95% CI = [9.1–11.6]. The confidence intervals indicate that both metabolites could be relatively reliably measured. The correlation between the levels of Glx and NAA was 0.65. There was a significant, negative association of age with NAA (r = −0.46, p < 0.0005) and Glx (r = −0.33, p < 0.0005) levels. The average (SD) signal to noise ratio (S/N) of the spectra was 21.3 (3.6), and de average Full Width Half Max (FWHM) was 0.041 (0.013). There was also a negative association between NAA and GM (r = −0.19, p = 0.024) and CSF (r = −0.19, p = 0.020).

The ANOVA showed a significant effect of group on both Glx (*F* (3, 139) = 3.6, *p *= 0.015) and NAA (*F* (3, 139) = 3.9, *p *= 0.011) levels. Post-hoc tests indicated that chronically ill patients had significantly lower levels of both metabolites than controls *p* < 0.05. However, this effect disappeared after adding the covariates in the ANCOVA due to a relation of age with phase of illness. Removing age as a covariate restored the significant association (p = 0.023 for Glx and p = 0.014 for NAA).

**Figure 2:**
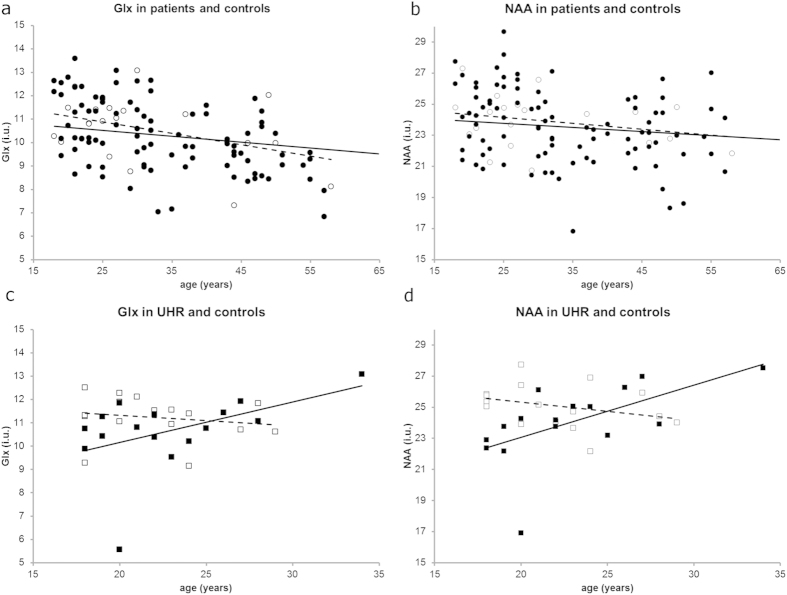
Linear regression of metabolite levels with age, (**a**) Glx and (**b**) NAA in patients (○) and (**c**) Glx and (**d**) NAA in UHR (□; dashed lines) compared to their control groups (• and ■ resp., solid lines).

Because of its relation with metabolite levels and because of its strong relation with duration of illness, the effect of age was investigated in patients and UHR with their corresponding healthy subgroups (UHR study had a separate control group) in supplementary analyses. Figure 2 shows the association of Glx and NAA with age for the different study groups separately. Supplementary Table S1 gives an overview of the regression analyses. In patients, there was no significant effect of age on both Glx and NAA levels and no effect of group (patient or control). Removing group and the group*age interaction from the model changed the effect for age to *p* < 0.0005 for Glx and *p* = 0.001 for NAA (not shown in table), indicating that group had a non-significant effect on the decline in metabolite level by age. In UHR, there was a significant effect of group (UHR vs. control; *p* = 0.013) and interaction of age*group (*p* = 0.022) for NAA and a trend for similar effects in Glx (group: *p* = 0.062, interaction: *p* = 0.088).

**Figure 3:**
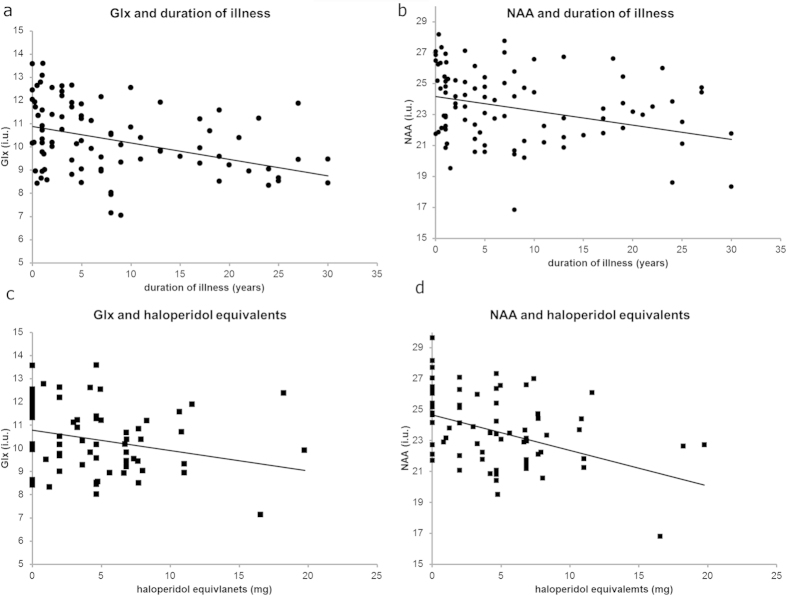
Linear regression of metabolite levels with duration of illness and haloperidol equivalents. (**a**) Glx and (**b**) NAA with duration of illness (•) and (**c**) Glx and (**d**) NAA with haloperidol equivalents (■) in patients (both recent onset and chronic).

**Figure 4:**
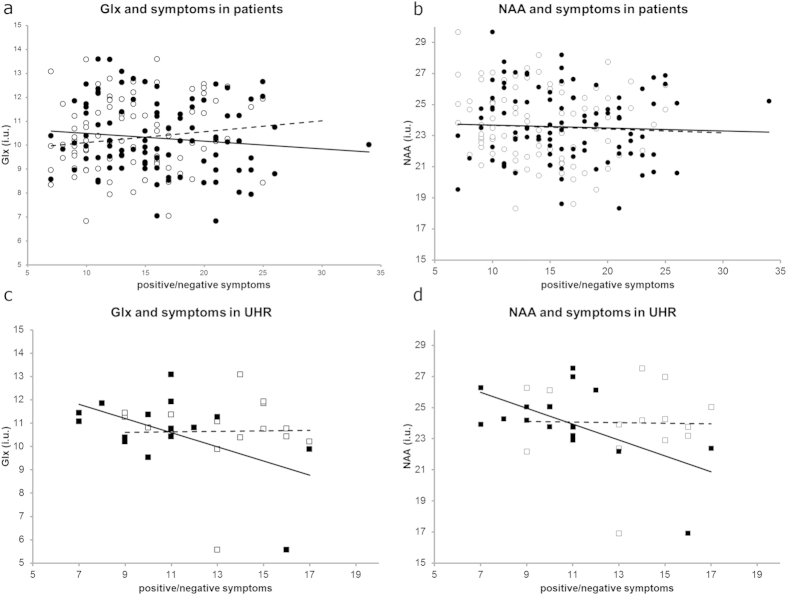
Linear regression of metabolite levels with positive symptoms and negative symptoms. (**a**) Glx and (**b**) NAA in patients and (**c**) Glx and (**d**) NAA in UHR; ○ (dashed line) = positive symptoms and • (solid line) = negative symptoms in patients, □ (dashed line) = positive and ■ (solid line) = negative symptoms in UHR.

Figure 3 shows the association between Glx and NAA levels and duration of illness/haloperidol equivalents in patients. Supplementary Table S2 gives an overview of the results of the regression analyses. There was a significant effect of duration of illness with both Glx (*p* = 0.003) and NAA (*p* = 0.035). There was no significant correlation between haloperidol equivalents and Glx or NAA levels. The effects of symptoms are shown in Fig. 4 and Supplementary Table S3. There was no significant association between symptoms and metabolite levels in both patients and UHR. When age was removed from the model, negative symptoms showed a (trend for a) significant negative correlation with Glx (*p* = 0.070) and NAA (*p* = 0.015) in UHR.

## Discussion

The most notable finding of the current study is that chronicity of schizophrenia was related to decreased levels of Glx and NAA. On the other hand, although on an exploratory note, UHR showed increased levels of prefrontal Glx and NAA with increasing age.

In patients, chronicity was related to lower levels of Glx and NAA, if no correction for age was applied. Moreover, the level of Glx and NAA were negatively related to duration of illness and there was a non-significant stronger decline in Glx and NAA with age in patients. These findings are in line with a meta-analysis showing that glutamate and NAA had a stronger decline (but glutamine a stronger increase) with age in patients than in healthy controls^12^, and with reviews showing some evidence for lower Glx and NAA levels in chronically ill schizophrenia patients^16^,^17^ and lower NAA levels in patients with earlier onset of illness^19^.

Moreover, we showed that Glx and NAA increased with age in our UHR sample, although this association should be treated with caution given small, on average young sample. In adolescents who have a high risk to transition to psychosis, excessive levels of glutamate may be present in the prefrontal cortex, as suggested by ^1^H-MRS studies and preclinical data^14^,^35^. In contrast to our study, one meta-analysis did not find differences between UHR and controls^19^ and another observed lower levels of NAA that were most apparent in younger subjects^21^. We also observed lower NAA levels in our youngest subjects, but NAA levels were higher than in controls after an age of 25 years. Of note, Brugger *et al.*^19^ included patients with a higher age and the meta-analyses also included subjects with a genetic risk for psychosis and Glx and NAA ratios relative to creatine. However, creatine levels increased with age in our UHR sample (not in controls, data not shown). It may thus be desirable to use absolute levels instead of ratios.

Taking these findings and the lower metabolite levels in the chronic sample together, our findings may support the NMDA-hypofunction hypothesis; early phases of the illness may be characterized with a toxic increase in glutamate levels^14^. These toxic glutamate levels, caused by N-Methyl-D-aspartate (NMDA) receptor dysfunction, may lead to white matter tissue loss and eventually decreased glutamate levels in chronic stages^35^,^36^. Concurrent NAA changes may also reflect this toxic glutamate increase, because these substances are biochemically linked^37^,^38^. One could also argue that the NAA signal primarily a marker of neuronal viability, and that changes in the NAA signal are a direct result of decreased brain volumes due to glutamate neurotoxicity. However, as CSF volumes did not show a significant effect, this explanation seems less plausible.

In schizophrenia patients, we failed to show a negative association between antipsychotic dosage and metabolite levels. Previous studies have shown that antipsychotic medication may both decrease and increase lateral frontal Glx levels^13^,^16^,^35^. In general, Glx levels are considered to appear unchanged in unmedicated patients^13^,^16^, although other studies do not support this view^35^. The huge variability in receptor profile of antipsychotics and possible interactions with factors such as duration of treatment may obscure the relation between antipsychotic treatment and metabolite levels.

A number of studies have shown a relationship between negative symptoms and Glx and NAA levels^13^,^16^,^19^. We observed (a trend for) a negative association between negative symptoms and Glx and NAA levels in UHR, again only without correcting for age. Given the strong correlation between age and negative symptoms (*r* = 0.52. *p* = 0.039), we speculate that negative symptoms may worsen during prodromal/early stages of the illness, concurrent with a decline in prefrontal glutamate possibly due to neurotoxicity.

There are a few limitations to this study. First, subjects were included from different studies with different objectives and the ^1^H-MRS scan was only part of the study protocol. However, only baseline scans before any treatment were included, minimizing study differences. Next, age was intrinsically correlated with both metabolite levels and subject characteristics, and it may be challenging to determine their unique effects. Moreover, it would be favorable to have a larger, longitudinal UHR sample and transition to psychosis as outcome measure, as the sample was currently small and only cross-sectional. In our study Glx and NAA were treated as separate metabolites they may influence each other at lower field strengths. However, we acknowledge this and don’t consider their similar findings as two separate phenomena. Finally, glutamate and glutamine could not be reliably separated with the current protocol. In following studies we plan to use a protocol that is able to do so.

In conclusion, in psychotic disorders, the decline in NAA and Glx is related to duration of illness. Our findings also suggest that UHR may be characterized by an increase in Glx and NAA with age. This initial increase and subsequent decrease of NAA and Glx during illness progression may support the NMDA-hypofunction hypothesis.

## Additional Information

**How to cite this article**: Liemburg, E. *et al.* Prefrontal NAA and Glx Levels in Different Stages of Psychotic Disorders: a 3T ^1^H-MRS Study. *Sci. Rep.* **6**, 21873; doi: 10.1038/srep21873 (2016).

## Supplementary Material

Supplementary Information

## Figures and Tables

**Table 1:** Demographical data.

	Chronic patients N = 60	Recent onset patients N = 31	UHR N = 16	Controls N = 36	p-value
Age	37.7 (10.5)	26.5 (8.8)	22.9 (4.3)	27.1 (10.3)	<0.0005^a^
Gender (% male)	80	71	56	66	0.19^b^
Education^d^	4.7 (1.8)	5.1 (1.6)	5.3 (1.4)	5.7 (0.9)	0.22^a^
Diagnosis					–
Schizophrenia	95.4%	67.7%			
Schizoaffective disorder	1.5%	3.2%			
Schizopreniform	0%	6.5%			
Psychotic disorder	1.5%	19.4%			
Delusional disorder	1.5%	3.2%			
Duration of illness (years)	12.4 (8.1)	0.8 (0.6)			–
Antipsychotic treatment (%, mean daily dose in mg)^d^					–
Antipsychotic free	7.7%	35.5%	100%		
Aripiprazole	21.6%, 13.6 mg	6.4%, 10 mg			
Clozapine	27.7%, 416.2 mg	3.2%, 150 mg			
Flupentixol	3.0%, 11.1 mg	0%			
Haloperidol	1.5%, 5 mg	3.2%, 3 mg			
Olanzapine	29.3%, 10.1 mg	35.5%, 8.2 mg			
Paliperidone	1.5%, 3 mg	0%			
Pimozide	0%	3.2%, 1 mg			
Quetiapine	6.2%, 375 mg	6.5%, 225 mg			
Risperidone	10.8%, 3.9 mg	6.5%, 5 mg			
Zuclopentixol	3.0%, 10.7 mg	3.2%			
Haloperidol equivalents (mg)	6.7 (7.1)	2.7 (3.2)			0.12^c^
PANSS positive	14.4 (4.5)	14.2 (5.3)	12.7 (2.4)		0.44^a^
PANSS negative	16.1 (5.1)	17.1 (5.5)	10.9 (2.9)		<0.0005^a^
PANSS general	31.9 (6.7)	31.2 (6.8)	27.1 (5.3)		0.076^a^

Overview of the characteristics of both patients groups, UHR subjects and the healthy control group.

^a^Kruskal Wallis test.

^b^Chi-square test for independence.

^c^Mann Whitney U test.

^d^Level according to the Verhage^31^ system.

^e^Part of the sample used 2 antipsychotics, standard deviations not given due to low numbers per cell.
