# Supplementary material for: Prefrontal NAA and Glx Levels in Different Stages of Psychotic Disorders: a 3T 1H-MRS Study
Source: Sci Rep. 2016 Feb 23;6:21873. doi: 10.1038/srep21873 (PMC4763193; doi:10.1038/srep21873)
Supplement: Supplementary Information [file srep21873-s1.pdf]

Supplementary information

Prefrontal NAA and Glx levels in different stages of a psychotic disorder: a 3T H-MRS study

Edith Liemburg[a,b,c,*],PhD, Anita Sibeijn-Kuiper[a], BSc, Leonie Bais[a,c], MSc, Gerdina Pijnenborg[d,e], PhD, Henderikus Knegtering[a,b,c], PhD, Jorien van der Velde[a], PhD, Esther Opmeer[a], PhD, Annerieke de Vos[a,e], MSc, Jozarni Dlabac-De Lange[a], MSc, Lex Wunderink[b,f], PhD, André Aleman[a,d], PhD

Supplementary Figure S1. Representative illustration of voxel placement in the left prefrontal cortex (radiological display) and below a representative resulting spectrum

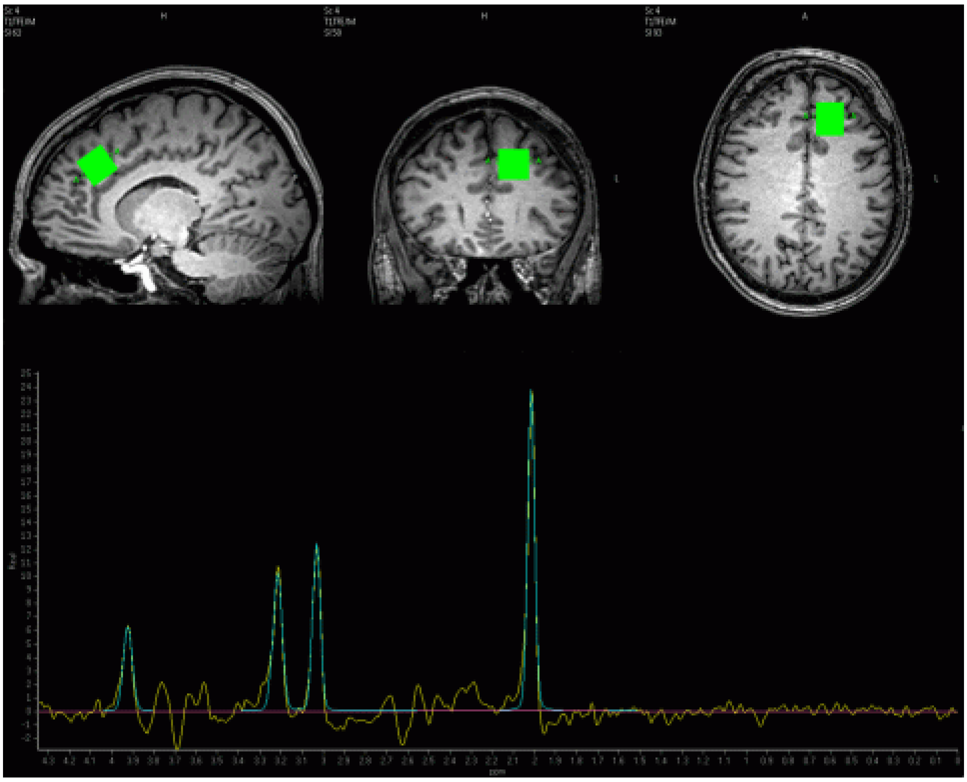

Supplementary Table S1 Regression of metabolite concentration against group (patients or UHR vs. healthy controls) with covariates (age, GM, CSF)

	B	Std. Error	β	t	Sig.
**Patients Glx**					
group	0.537	10.026	0.128	0.523	0.602
age	-0.049	0.027	-0.354	-10.783	0.077
age*group	-0.021	0.030	-0.220	-0.698	0.487
GM	-0.027	20.116	-0.001	-0.013	0.990
CSF	-40.052	60.153	-0.077	-0.659	0.512
**Patients NAA**					
group	0.375	10.732	0.055	0.216	0.829
age	-0.053	0.046	-0.239	-10.154	0.251
age*group	-0.021	0.051	-0.135	-0.410	0.682
GM	-40.794	30.571	-0.160	-10.342	0.182
CSF	-120.602	100.387	-0.148	-10.213	0.228
**UHR Glx**					
group	-50.404	20.766	-20.065	-10.954	0.062
age	-0.026	0.093	-0.077	-0.276	0.784
age*group	0.214	0.121	10.930	10.771	0.088
GM	10.180	20.957	0.081	0.399	0.693
CSF	50.136	40.948	0.212	10.038	0.309
**UHR NAA**					
group	-100.731	40.034	-20.674	-20.661	0.013
age	-0.098	0.136	-0.189	-0.718	0.479
age*group	0.429	0.176	20.528	20.439	0.022
GM	-10.798	40.313	-0.080	-0.417	0.680
CSF	30.181	70.216	0.085	0.441	0.663

Supplementary Table S2 Regression of metabolite concentration against duration of illness and haloperidol equivalents with covariates (age, GM, CSF)

	B	Std. Error	Beta	t	Sig.
**Duration of illness Glx**					
Duration of illness	-0.061	0.020	-0.317	-3.063	0.003
GM	2.469	2.474	0.123	0.998	0.321
CSF	-11.288	6.712	-0.216	-1.682	0.096
**Duration of illness NAA**					
Duration of illness	-0.072	0.033	-0.226	-2.143	0.035
GM	-0.725	4.149	-0.022	-0.175	0.862
CSF	-18.144	11.257	-0.211	-1.612	0.111
**Haloperidol equivalents Glx**					
Haloperidol equivalents	-0.136	0.094	-0.198	-1.453	0.153
age	-0.058	0.038	-0.224	-1.538	0.131
GM	-2.695	6.161	-0.078	-0.437	0.664
CSF	-2.585	14.585	-0.031	-0.177	0.860
**Haloperidol equivalents NAA**					
Haloperidol equivalents	-0.036	0.050	-0.089	-.718	0.476
Age	-0.071	0.020	-0.464	-3.495	0.001
GM	0.491	3.321	0.024	0.148	0.883
CSF	-4.957	7.863	-0.101	-0.630	0.531

Supplementary Table S3 Regression of metabolite concentration symptoms in both patients and UHR with covariates (age, GM, CSF)

	B	Std. Error	β	t	Sig.
**Patients Glx**					
Positive symptoms	0.037	0.034	0.103	10.103	0.273
Negative symptoms	-0.040	0.028	-0.131	-10.440	0.154
age	-0.068	0.013	-0.488	-50.032	<0.0005
GM	0.278	20.239	0.015	0.124	0.901
CSF	-70.234	60.264	-0.143	-10.155	0.251
**Patients NAA**					
Positive symptoms	-0.012	0.059	-0.019	-0.197	0.844
Negative symptoms	-0.025	0.048	-0.050	-0.515	0.608
age	-0.077	0.024	-0.335	-30.262	0.002
GM	-60.758	30.918	-0.218	-10.725	0.088
CSF	-100.432	100.962	-0.125	-0.952	0.344
**UHR Glx**					
Positive symptoms	-0.056	0.168	-0.091	-0.334	0.745
Negative symptoms	-0.191	0.179	-0.330	-10.065	0.312
age	0.124	0.113	0.334	10.100	0.297
GM	-30.293	60.539	-0.174	-0.504	0.625
CSF	100.830	150.891	0.244	0.682	0.511
**UHR NAA**					
Positive symptoms	-0.092	0.229	-0.097	-0.401	0.697
Negative symptoms	-0.418	0.244	-0.472	-10.714	0.117
age	0.196	0.154	0.344	10.273	0.232
GM	-40.045	80.924	-0.139	-0.453	0.660
CSF	-40.689	210.686	-0.069	-0.216	0.833
